# Epigenomic Modifications Predict Active Promoters and Gene Structure in Toxoplasma gondii

**DOI:** 10.1371/journal.ppat.0030077

**Published:** 2007-06-08

**Authors:** Mathieu Gissot, Krystyna A Kelly, James W Ajioka, John M Greally, Kami Kim

**Affiliations:** 1 Department of Medicine (Infectious Diseases), Albert Einstein College of Medicine, Bronx, New York, United States of America; 2 Department of Microbiology and Immunology, Albert Einstein College of Medicine, Bronx, New York, United States of America; 3 Department of Pathology, University of Cambridge, Cambridge, United Kingdom; 4 Medical Research Council Biostatistics Unit, Institute of Public Health, Cambridge, United Kingdom; 5 Department of Medicine (Hematology), Albert Einstein College of Medicine, Bronx, New York, United States of America; 6 Department of Molecular Genetics, Albert Einstein College of Medicine, Bronx, New York, United States of America; Institut Pasteur, France

## Abstract

Mechanisms of gene regulation are poorly understood in Apicomplexa, a phylum that encompasses deadly human pathogens like *Plasmodium* and *Toxoplasma*. Initial studies suggest that epigenetic phenomena, including histone modifications and chromatin remodeling, have a profound effect upon gene expression and expression of virulence traits. Using the model organism *Toxoplasma gondii,* we characterized the epigenetic organization and transcription patterns of a contiguous 1% of the T. gondii genome using custom oligonucleotide microarrays. We show that methylation and acetylation of histones H3 and H4 are landmarks of active promoters in T. gondii that allow us to deduce the position and directionality of gene promoters with >95% accuracy. These histone methylation and acetylation “activation” marks are strongly associated with gene expression. We also demonstrate that the pattern of histone H3 arginine methylation distinguishes certain promoters, illustrating the complexity of the histone modification machinery in *Toxoplasma.* By integrating epigenetic data, gene prediction analysis, and gene expression data from the tachyzoite stage, we illustrate feasibility of creating an epigenomic map of T. gondii tachyzoite gene expression. Further, we illustrate the utility of the epigenomic map to empirically and biologically annotate the genome and show that this approach enables identification of previously unknown genes. Thus, our epigenomics approach provides novel insights into regulation of gene expression in the Apicomplexa. In addition, with its compact genome, genetic tractability, and discrete life cycle stages, T. gondii provides an important new model to study the evolutionarily conserved components of the histone code.

## Author Summary

Apicomplexan parasites, including *Toxoplasma gondii,* are responsible for a variety of deadly infections, but little is understood about how these important pathogens regulate gene expression. Initial studies suggest that alterations in chromatin structure regulate expression of virulence traits. To understand the relationship of chromatin remodeling and transcriptional regulation in *T. gondii,* we characterized the histone modifications and gene expression of a contiguous 1% of the T. gondii genome using custom DNA oligonucleotide microarrays. We found that active promoters have a characteristic pattern of histone modifications that correlates strongly with active gene expression in tachyzoites. These data, integrated with prior gene predictions, enable more accurate annotation of the genome and discovery of new genes. Further, these studies illustrate the power of an integrated epigenomic approach to illuminate the role of the “histone code” in regulation of gene expression in the Apicomplexa.

## Introduction

Toxoplasma gondii is an obligate intracellular apicomplexan parasite responsible for encephalitis in immunocompromised individuals and birth defects when a fetus is exposed in utero [1,2]. The life cycle of T. gondii is complex, with multiple differentiation steps that are critical to survival of the parasite in its human and feline hosts [3]. The genetic tractability of T. gondii has caused it to emerge as a model for the study of apicomplexan parasites [3], and the recent sequencing of the T. gondii genome (http://www.toxodb.org) is adding to our appreciation of the unusual nature of apicomplexan genomes [4,5].

A remarkable finding is the relative paucity of genes encoding proteins with motifs that indicate transcription factor function in apicomplexan genomes [6,7]. This has led to the proposal that gene regulation in apicomplexan parasites is controlled mainly via RNA stability [6], despite the tightly regulated patterns of gene expression observed in different stages of the life cycle of T. gondii [8] and Plasmodium falciparum [9]. However, that certain DNA motifs are recurrent in the promoters of these organisms and bind to nuclear factors [10−14] suggests that unrecognized transcription factors may exist, but are not encoded by genes with recognizable structural features. On the other hand, the RNA polymerase II machinery [7,15] and genes with motifs indicating potential chromatin remodeling and modification functions [6,16] are conserved within the Apicomplexa. Epigenetic processes have significant clinical relevance in light of studies that implicate the histone deacetylase Sir2 homolog in regulation of antigenic variation in P. falciparum [17,18].

To obtain a genome-wide view of gene expression in T. gondii tachyzoites, we examined the epigenetic organization and transcription patterns of a contiguous 1% of the T. gondii genome using custom microarrays. Histone modifications—including acetylation of histone H4 (H4ac), acetylation of lysine 9 (H3K9ac), and trimethylation of lysine 4 of histone H3 (H3K4me3)—have been identified at certain individual active loci in T. gondii [19], suggesting a role in gene expression. We hybridized the tiled genomic microarrays with material derived from chromatin immunoprecipitations using antibodies to modified histones. By simultaneously hybridizing the microarray to tachyzoite-derived cDNA, we tested the genome-wide association of specific histone modifications with gene expression.

## Results

### Microarray Design and Experimental Scheme

**Figure 1:**
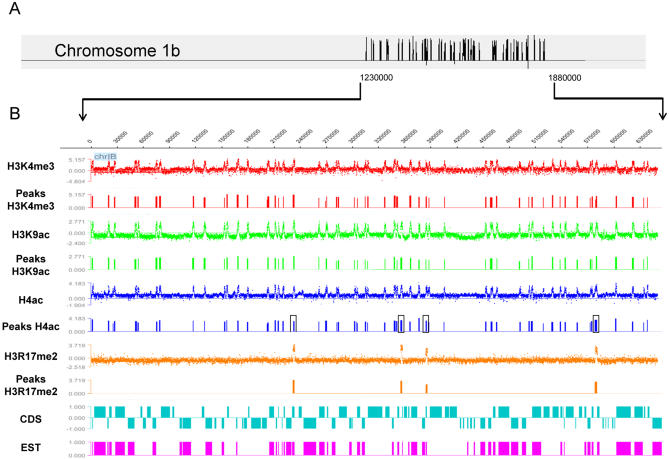
ChIP-on-Chip Experiments Using “Activation” Histone Markers (A) Genomic view of Chromosome 1b. Linear representation of Chromosome 1b (2 Mb) with the position of the 650,000-bp region tiled on the NimbleGen chip. The positions of high significance H3K9ac peaks are indicated with vertical lines. The chip encompassed the region from 1,230,000 to 1,880,000 bp of Chromosome 1b of the T. gondii RH strain. Sequences of the tiled oligonucleotides can be obtained from the Gene Expression Omnibus database (see Supporting Information). (B) ChIP-on-chip hybridization. Paired tracings of raw data (top tracing) and the distribution of peaks (lower tracing) for “activation” marks H3K4me3 (red), H3K9ac (green), H4ac (blue), and H3R17me2 (orange) within a 650-kb region of Chromosome 1b. Data are presented as the log_2_ ratio of the hybridization signal given by DNA immunoprecipitated using the indicated antibody compared with the signal given by the input DNA sample. The positions of the 91 predicted genes (CDS, light blue bars; from start to stop codons; up = sense; down = antisense) and tachyzoite ESTs (purple) are presented. The scale is indicated on the left side of the figure and the position on the sequence at the top. H4ac peaks corresponding to the H3R17me2 peaks are boxed. Genomic positions correspond to the region of Chromosome 1b investigated (position 0 in the region is position 1,230,000 in Chromosome 1b).

We generated a custom oligonucleotide microarray containing 12,995 50-mer features tiling a 650-kb region of Chromosome 1b, with an average resolution of one oligonucleotide every 50 bp (Figure 1). Chromosome 1b of the RH strain of the 63-Mb T. gondii genome has been extensively annotated and has a single nucleotide polymorphism frequency comparable with the rest of the genome, an average of 5.7 exons per coding sequence (CDS), and a gene density of one gene per 7.4 kb [20]. Currently, 91 genes are predicted within the 650-kb region of the RH strain of T. gondii.

The amino acid sequences of the tails of eukaryotic histones H3 and H4 are strongly conserved (Figure S1), allowing us to use a panel of commercial antibodies for chromatin immunoprecipitation (ChIP) in T. gondii. After screening antibodies to modified histones for T. gondii nuclear localization (Table 1; Figure S2), we performed ChIP using DNA isolated from the intracellular tachyzoite stage of T. gondii. As a control, we used an antibody against a T. gondii kinase with no DNA-binding potential (M. Gissot and K. Kim, unpublished data). The immunoprecipitated DNA was amplified, tested to ensure enrichment for control loci was maintained, and co-hybridized to the 650-kb tiling array with input DNA.

**Table 1:**
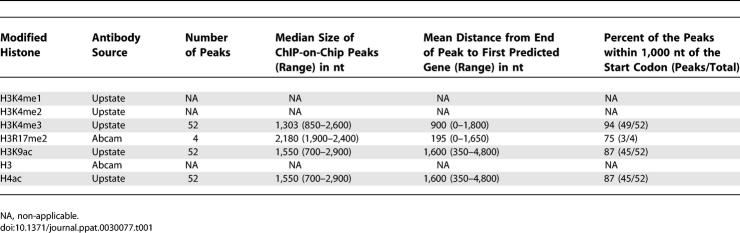
Distribution of Modified Histones Tested in ChIP-on-Chip Assays

### Modified Histones H4ac, H3K9ac, and H3K4me3 Are Enriched at Promoter Regions

We studied the distribution of three modified histones (H4ac, H3K9ac, and H3K4me3) previously described as activation marks in other eukaryotes [21,22]. The ChIP material applied to the microarray (ChIP-chip) generated strong focal peaks of enrichment for the three different modified histones. Signal was readily discriminated from noise, even looking at the raw ChIP/input DNA ratio (Figure 1), a finding confirmed by the *p*-values derived from the use of the ChIPOTle analytical approach [23], which approached zero for each locus. We observed 52 clear, discrete, and coincident H4ac, H3K9ac, and H3K4me3 peaks within the 650-kb region tiled on the microarray (Figure 1B; Table S1). The H3K9ac and H4ac peaks have a median size of 1,550 bp, whereas the H3K4me3 peaks are relatively smaller (median size of 1,300 bp; Table 1).

As previously observed for other eukaryotes [21,22], these modifications co-localize and associate to form a complex pattern at focused loci in the T. gondii genome (Figure 1). More than 96% of the H3K4me3, H3K9ac, and H4ac (Figure 1B) peaks are placed in the predicted intergenic regions. Moreover, the identified peaks for the three modifications are located close to the 5′ of predicted genes. Indeed, the distance between the identified peak and the start codon of the closest gene is less than 1,000 bp for more than 85% (45/52) of the H3K9ac and H4ac peaks (Table 1), and less than 1,500 bp for more than 90% (48/52). Similarly, for more than 90% (49/52) of H3K4me3 peaks, the end of the peak and the first predicted initiation codon was within 1,000 bp (Table 1).

### Modified Histone H3R17me2 Is Enriched at a Subset of Promoter Regions, but H3K4me1 and H3K4me2 Are Not Enriched at Promoters

We also performed ChIP using antibodies against histone H3 dimethylated at arginine 17 (H3R17me2), another putative general activation mark in T. gondii [19] and other eukaryotes [24,25]. Recently, it was suggested that this histone modification is present at all active promoters in T. gondii [19] based upon PCR examination of selected promoters after ChIP with anti-H3R17me2. Using the same antibody, we show that this modification is restricted to a subset of promoters (Figure 1B). This histone mark overlapped with only four of the 52 modified histone peaks identified (4.5% of the genes present on the microarray). All four genes have expressed sequence tags (ESTs) for both tachyzoite and bradyzoite stages.

The H3K4me1 and H3K4me2 marks were also investigated using ChIP-chip and were not specifically enriched, as determined by analysis of hybridizations by the ChIPOTLe software (unpublished data). We also verified that the modified histone peaks identified were not due to local core histone enrichment by performing ChIP-chip with an antibody specific to the C-terminus histone H3.

### ChIP Validation of Genome Array Hybridization Results

**Figure 2:**
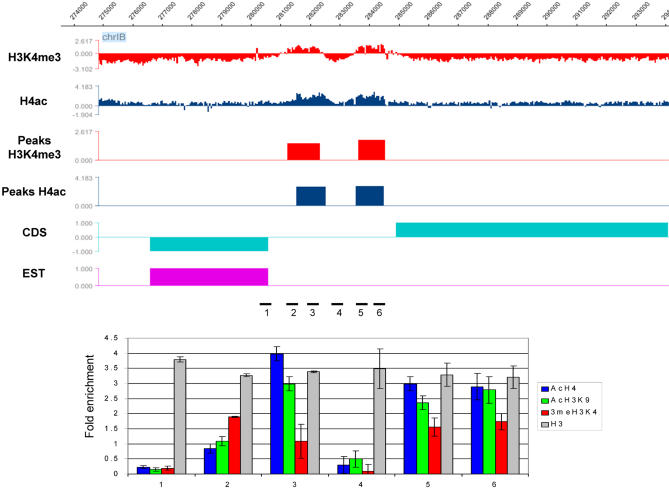
Validation of ChIP-on-Chip Results with ChIP and Real-Time Quantitative PCR The raw data and the distribution of peaks identified for H3K4me3 (red) and H4ac (blue) are presented for one specific locus. H3K9ac peaks (not shown; Table 1 and Figure 2) have nearly identical distribution to H4ac peaks. The predicted genes (light blue) are presented at the bottom of the figure together with the EST data (purple). The scale is indicated on the left side of the figure and the position on the sequence at the top. Real-time quantitative PCR verification of the ChIP with the indicated antibodies was performed using primers amplifying the six regions indicated by the bars. The H3K4me3 peaks are shifted toward the 5′ end of the gene, indicating the orientation of transcription (compare primer sets 2 and 3). Enrichment was not significantly changed when the DNA was immunoprecipitated using an anti-H3 antibody, indicating hybridization peaks are not due to localized nonspecific enrichment of histones.

The ChIP-chip results were validated using quantitative single-locus PCR (Figure 2 is representative of eight loci validated). Using real-time quantitative PCR on ChIP samples, we amplified regions within the predicted gene (primer set 1) or in intergenic sequences (primer sets 2−6). We found enrichment of the three modified histones in regions identified as peaks in the ChIP-chip experiments (primer sets 2, 3, 5, and 6). In contrast, the three activation marks tested were not significantly enriched in a region located within the predicted gene (primer set 1) and a region between the two identified peaks (primer set 4). We also verified that there was no significant local enrichment of the core histone H3 (positive PCR for all primer sets).

### Distribution of Modified Histone Peaks Correlates with Gene Expression

**Figure 3:**
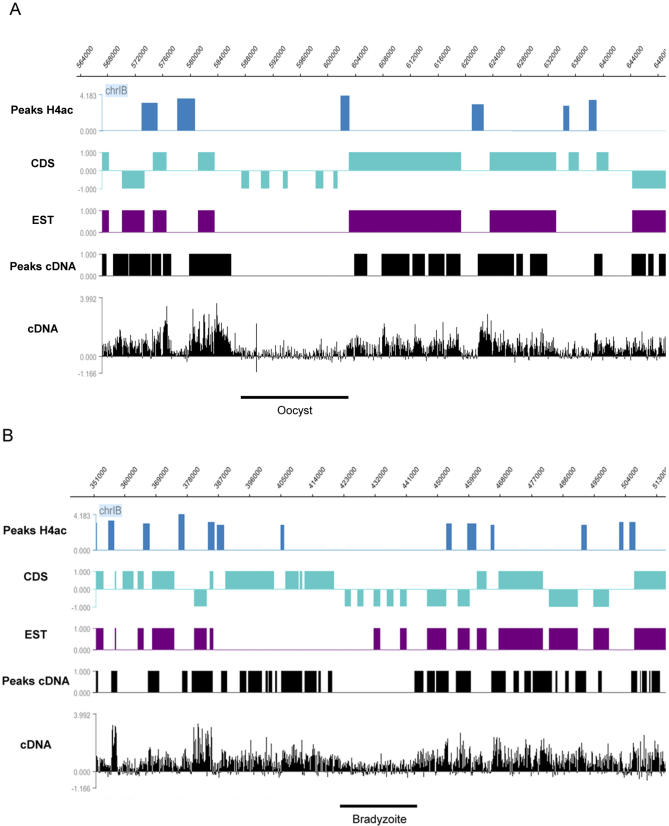
Gene Expression Correlates with the Enrichment of “Activation” Histone Modifications and EST Mapping Distribution of identified peaks for H4ac (blue) is presented as shown for two regions of Chromosome 1b (A, B). The raw data (cDNA hybridization signal) and significant peaks of expression are shown in black. The predicted genes are represented by the light blue boxes (up = sense; down = antisense) and positions of genes with at least one tachyzoite EST are shown in purple. Two regions of clustered stage-specific genes that lack gene expression and modified histone peaks are underscored by a black bar ([A] oocyst genes, [B] bradyzoite genes).

To verify the link between the modified histone peaks and transcription of the nearest predicted gene, we hybridized cDNA made from intracellular tachyzoites to the tiled microarray (Figure 3). Using three different analytical approaches, we identified regions on the tiled portion of the genome with significant gene expression (Table S1). Overall, 51 of the 52 regions with a cluster of H3K9ac, H4ac, and H3K4me3 peaks had a significant cDNA hybridization signal adjacent to them. These data were consistent with EST studies, with 46 of the 49 genes represented by at least one EST for the tachyzoite stage expressed in our dataset. In our study, 31% (21/67) of the genes expressed were not represented by any EST data, demonstrating the limits of the EST mapping approach for identifying expressed genes.

Two transcribed loci did not correspond to a predicted gene (Table S1). One locus had associated H3K9ac, H4ac, and H3K4me3 peaks characteristic of active chromatin and corresponded to a transcription unit represented by two overlapping tachyzoite ESTs (CN197705 and CK737836). A partial open reading frame (ORF) was discovered after alignment of those ESTs. After comparing this sequence with the nr database (http://www.ncbi.nlm.nih.gov/BLAST), we found the ORF had homology with the cytochrome oxidase subunit III (COX3) gene of *Plasmodium* (highest *p*-value = 5e-06). This gene is not annotated in the current version of the T. gondii genome (http://www.toxodb.org). The other locus was also represented by an EST (BG659482) and appears to be driven by a promoter that displays promoter activity in both directions (Table 2). However, this transcribed locus does not have an ORF and appears to represent a non-coding RNA.

**Table 2:**
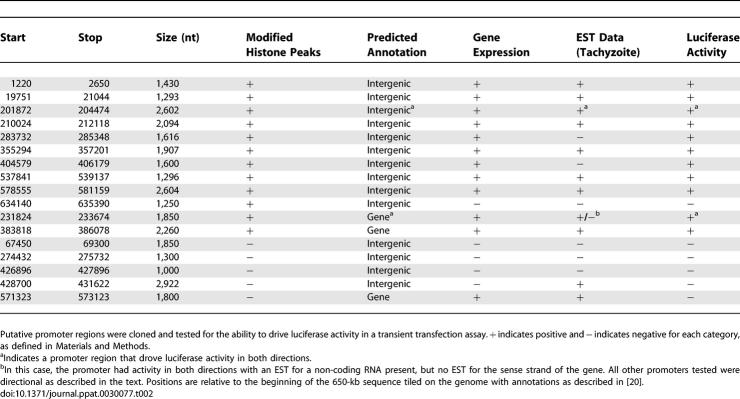
Promoter Activities and Gene Expression of Selected Putative Promoters

We also found two regions of clustered genes with stage-specific expression based on EST data. One region predicts a set of five tandemly arrayed kinases with ESTs primarily from the oocyst stage (Figure 3A). Another region is characterized by five genes predicted as BSR4 homologues with ESTs primarily from the bradyzoite stage (Figure 3B). No significant expression during the tachyzoite stage could be detected, and neither region had any of the three histone modification peaks characteristic of active chromatin. For the bradyzoite-specific locus, two ESTs were recovered from a Type III strain (VEG) tachyzoite cDNA library. These ESTs could reflect differences in gene expression between strains or represent the low level of bradyzoite forms frequently present in Type II and Type III tachyzoite cultures.

### Regions Encompassing H4ac and H3K9ac Peaks Are Able to Drive Luciferase Expression

**Figure 4:**
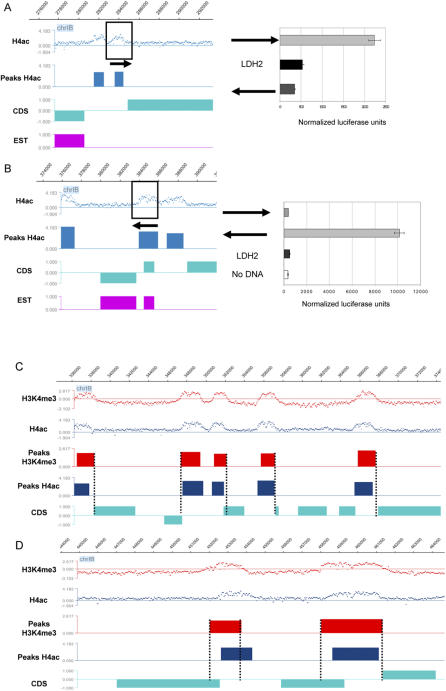
Promoter Activity of Regions with Histone Modifications (A, B) Predicted promoters drive luciferase expression. Two regions with H4ac peaks (blue) and the position of annotated genes (light blue) are shown (left). Positions of tachyzoite-specific ESTs are shown in purple. (A) Shows activity for a gene without a predicted EST but with cDNA hybridization. (B) Shows promoter activity for a peak that lies within a predicted gene coding region but can drive expression of a gene transcribed on the opposite strand, as determined by EST data and cDNA hybridization. The regions that tested the luciferase reporter assay are boxed. Each sequence was cloned in the sense and antisense direction (arrows) and showed directional activity. The graph represents Firefly luciferase units normalized to the Renilla luciferase units of the co-transfected constitutive tubulin promoter. Controls included untransfected parasites (no DNA) or parasites transfected with the Firefly luciferase reporter under the control of LDH2, a bradyzoite-specific promoter not expressed at this stage of the life cycle [39]. (C, D) H3K4me3 peaks are shifted toward the 5′ end of genes. Raw data and the distribution of H3K4me3 (red) and H4ac (blue) peaks are presented. H3K9ac peaks (not shown) have nearly identical distribution to H4ac peaks. The predicted genes (CDS) are represented by the light blue boxes (up = sense; down = antisense). The H3K4me3 peaks are shifted to the 5′ end of genes relative to the H4ac peaks (Positions of the ends of H3K4me3 peaks are illustrated with a dotted line to facilitate comparison). The scale is indicated on the left side of the figure and the position on the sequence at the top. (D) Also shows a broad H3K4me3 peak that encompasses an H4ac peak, which corresponds to an intergenic region that displays promoter activity in both directions.

To test that the clustered peaks were located at active promoters, we performed luciferase reporter assays (Table 2; Figure 4A and 4B). We cloned regions spanned by the H3K9ac and H4ac peaks and tested their ability to drive the expression of the luciferase in transient transfection assays. Of the 12 loci tested, 11 were able to drive expression of luciferase (Table 2; Figures 4 and S3).

Regions 5′ of non-expressed genes that lacked clustered peaks of modified histones or regions spanned by a predicted ORF are not able to drive the expression of luciferase (Table 2; Figure S3). However, the two loci with overlapping H3K4me3, H3K9ac, and H4ac peaks located within rather than 5′ to annotated gene coding regions were both able to drive the expression of a reporter gene (Table 2; Figure 4B).

Of the 52 activation peaks identified, only one lacked evidence of mRNA expression in its vicinity. This peak is located 5′ to a predicted gene (Tg1b.2420), a locus with the characteristics of a DNA-repair protein, but not associated with any EST in the T. gondii database at any stage of the life cycle. The promoter of this gene yielded a background activity as low as the untransfected parasites (Table 2; Figure S3).

### The H3K4me3 Mark Predicts Directionality of the Promoter

H3K4me3 peak distribution is consistently shifted toward the 5′ end of genes in comparison with H3K9ac (unpublished data) and H4ac peaks (Figure 4C and 4D). PCR studies confirmed that the shift of the H3K4me3 peak predicts the orientation of transcription. (Four genes were tested with two represented in Figure 2.) As predicted by these data, most of the sequences tested have directional activity, as would be expected for genuine promoters (Table 2; Figure 4C and 4D). However, seven of the 52 peaks are located in regions where two genes are transcribed in opposite directions, providing biological evidence for sequences in T. gondii that have promoter activity in both directions as shown in other Apicomplexa [26].

### Integration of ChIP-chip and Gene Expression Data Improves Annotation of the Genome

As illustrated, H3K4me3, H3K9ac, and H4ac peaks identify promoters. We also found seven predicted genes (as defined in [20]) that were expressed but lacked modified histone peaks at their predicted promoter. In all such cases, these genes are preceded within 1,000 bp by an expressed gene that is transcribed in the same direction and bears histone activation peaks at its 5′ end. These genes likely represent gene prediction errors, since RT-PCR in two cases confirmed a single transcription unit with the adjacent gene (Figure S4).

## Discussion

We have employed an integrative approach to epigenomics, combining simultaneous analysis of ChIP-on-chip and gene expression on a tiling array encompassing a 0.65-Mb contiguous portion of the T. gondii Chromosome 1b. The H3K9ac, H4ac, and H3K4me3 modifications co-localize at focused loci in the T. gondii genome and correlate with significant gene expression. We confirmed that the enrichment observed was not due to local enrichment of the H3 core histone by performing ChIP with an antibody directed against the C-terminus of the histone H3. In contrast, in *T. gondii,* the H3K4me1 and the H3K4me2 modifications are present at equal amounts in active and inactive chromatin as previously shown for human promoters [22] and in contrast to Saccharomyces cerevisiae [23].

To our knowledge, this study is the first to explore the distribution of the H3R17me2 modification on a genomic scale. Surprisingly, this modification is enriched only at a subset of active promoters. Thus, T. gondii uses its histone modification machinery not only as a general landmark of activated promoters but also to specifically attribute a distinctive mark to certain promoters. ESTs have been sequenced from both tachyzoite and bradyzoite stages for those four genes, whereas only 26 of the 91 predicted genes on our chip (28%) have ESTs in both tachyzoite and bradyzoite stages. The H3R17me2 mark may have significance during the tachyzoite to bradyzoite differentiation process, but the number of loci discovered in this study are too limited to speculate further upon the specificity conferred by this trait. The recent discovery of the importance of arginine methylation during early development of mouse embryo indicates a specific role for the H3R17me2 during differentiation [27].

The H3K9ac and H4ac peaks in T. gondii are larger than those previously observed in human (approximately 700 nucleotides [nt]) [28] but similar in size to those found in yeast [23]. It appears that the number of modified nucleosomes is in the same range for these three organisms despite their difference in genome compaction. Such similarity in the size of the peaks may have functional implications for RNA polymerase II.

The placement of the three “gene activation” modifications coincides, but H3K4me3 peaks are shifted toward the 5′ end of expressed genes. This difference has been documented in human cell lines [29] and predicts the directionality of promoters in T. gondii. Although most promoters appear to be orientation-specific, the tiled region of the T. gondii genome encodes several regions that exhibit promoter activity in both directions. Further mapping studies are needed to determine whether these are true bi-directional promoters or two separate promoters facing in opposite directions.

We observed an exceptional correlation between gene expression and the presence of co-localized modified histone peaks. The few discrepancies between the EST database and our gene expression data are likely due to differences in gene expression between the strain we used (RH, Type I) and the strains used to generate “tachyzoite” cDNA libraries. Type II and III tachyzoite cultures, in contrast to Type I strains, frequently have a low level of basal bradyzoite forms.

One region represented on our array had a cluster of H3K9ac, H4ac, and H3K4me3 peaks but was unable to drive luciferase expression. Interestingly, these peaks are located 5′ to a gene (Tg1b.2420) predicted to encode a protein similar to DNA-repair protein XRCC3, a protein essential for ultraviolet radiation–induced double-strand break repair from bacteria to mammals [30]. Expression of this gene was not detected by reverse transcriptase−PCR (RT-PCR; Table S1) and there were no associated ESTs in the T. gondii database at any stage of the life cycle, which could be explained by rapid processing or degradation of the mRNA for this gene. Alternatively, the promoter could be in a poised state waiting for activation or for the release of a repression, as observed in a study of rapidly induced genes in human T cells [31]. As suggested for T cells, the activation marks associated with this promoter could signify the presence of epigenetic memory in T. gondii. In a study of human promoters, 20% of those genes with overlapping H3ac and H3K4me3 marks lacked evidence of mRNA expression [21].

Prior microarray gene expression studies in T. gondii have been based upon cDNAs [32] rather than tiled genomic microarrays. Our survey of tachyzoite gene expression for this contiguous 1% of the T. gondii genome enabled us to identify new tachyzoite-expressed genes and discover transcripts in regions where genes have not been predicted. For example, a cluster of modified histone “activation” peaks helped us to identify a gene coding for a cytochrome oxidase subunit III, which is not annotated in the current version of the T. gondii genome, and a possible non-coding RNA. Moreover, our study illustrates the power of empirical annotation of the genome in terms of promoters and their transcriptional orientation, enhancing gene prediction approaches beyond what is currently possible using DNA sequence-based approaches alone.

In conclusion, we have performed the first mapping to our knowledge of the epigenome of an apicomplexan parasite. Taken together, the data indicate that T. gondii uses a multipart histone modification system to assign a functional role to certain DNA sequences and underscores the ability of this unicellular apicomplexan parasite to employ a complex set of tools to control its gene expression. These data are consistent with the extensive repertoire of proteins predicted to modify histones in the T. gondii genome [16]. Moreover, our study illustrates the power of empirical annotation of the genome in terms of promoters and their transcriptional orientation, enhancing gene prediction approaches beyond what is currently possible using DNA sequence-based approaches alone.

T. gondii is a medically important pathogen and is genetically tractable. It is a powerful model for studying the gene regulation of apicomplexan parasites and may now represent a new model system for understanding evolutionarily conserved components of the “histone code.” Further, epigenetic regulators may represent potential therapeutic targets and provide new tools to fight toxoplasmosis and other parasitic diseases like malaria.

## Materials and Methods

### Parasite culture.

T. gondii RH strain was maintained in confluent monolayers of human foreskin fibroblasts (HFF). Parasites were harvested 24 h after invasion of HFF cells and purified as previously described in [33].

### Chromatin immunoprecipitation (ChIP).

ChIP was performed as described [34] with slight modifications. Briefly, chromatin from intracellular tachyzoites grown in HFF for 24 h was cross-linked for 10 min with 1% formaldehyde at room temperature and purified after a sonication step yielding fragments of 500−1,000 bp. Immunoprecipitations were performed with the appropriate rabbit serum (Table 1) at 4 °C overnight and washed extensively as published previously [34]. DNA was further subjected to a treatment with proteinase K for 2 h and then purified using the Qiagen PCR purification kit (http://www.qiagen.com). As a negative control, we used rabbit antiserum to PKA2, a kinase that is not present in the nucleus (M. Gissot and K. Kim, unpublished data).

### Design of the Nimblegen oligonucleotide microarray and ChIP-on-chip.

We generated a tiled array of 50-bp oligonucleotides with 12,295 oligos encompassing 650,000 bp (1,230,000−1,880,000) of Chromosome 1b [20] with a spacing of 50 bp between each oligonucleotide. The array was fabricated in the NimbleGen Systems (http://www.nimblegen.com) 12-plex format, which allows simultaneous hybridization of 12 identical arrays on a single slide. Amplification of immunoprecipitated DNA and 100-ng input DNA was performed using the ligation-mediated PCR technique [35]. After amplification, the immunoprecipitated DNA was tested for enrichment of control loci by qPCR and co-hybridized to the 650-kb tiling array with input DNA. DNA was labeled using random primers coupled to a fluorochrome and hybridized according to NimbleGen Systems procedures. At least two biological replicates were performed.

### Real-time quantitative PCR.

Real-time quantitative PCR was performed on the 7300 ABI apparatus using the Power Sybr (ABI, http://www.appliedbiosystems.com) mastermix in a 20-μL volume according to the manufacturer's instructions. PCR primers were designed using the Primerexpress software (ABI) to amplify regions of 100−150 nt. A 10-fold dilution of input was compared with 0.5 ng of immunoprecipitated DNA. Each experiment was performed at least three times in duplicate.

### Tachyzoite gene expression.

The RNA from three replicate flasks containing RH strain–infected HFFs and one control flask containing only HFFs was purified using TRIzol. RNA integrity was verified on the Agilent Bioanalyzer (http://www.agilent.com). Ten micrograms of total RNA was retrotranscribed using the BD Sprint Powerscript kit (http://www.bdbiosciences.com) and random hexamers and made double-stranded cDNA (dscDNA) using Escherichia coli polymerase I. dscDNA labeling with fluorochrome-coupled random hexamer and hybridization to the array was performed following NimbleGen protocols. NimbleGen scanning and spot finding software were used.

### Statistical analysis of array data.

Significant peaks for ChIP-on-chip were identified with the ChIPOTle software [23] using a permutation simulation to estimate the background distribution (with a window size of 500 bp, 300 permutations, and a *p*-value of 0.001). Peaks with a *p*-value of less than 10^−10^ (which produces about 50 times more significant regions than false regions) and with a peak height cut-off of 2 were considered significant. The false discovery rate was 0.1%.

After background correction using random probes, gene expression was calculated as the average of the log_2_ ratio of the intensity given by the HFF plus parasite dscDNA to the intensity given by the HFF-alone dscDNA. With ChIPOTle, expression was considered significant with a *p*-value < 0.05 and a high average ratio above 1 or 0.6. Peaks of significant expression were also identified using the detection peaks tool in SignalMap software with a sliding window of 150 bp and a log_2_ ratio threshold of 1 or 0.8. A peak is identified when there are at least four data points within a window above the threshold value. The height of the peak is the maximum of the data points within the window. In addition, the raw log_2_ ratios were normalized using loess regression to remove the dependence of the variance on the mean and partitioned into segments along the chromosome with the function *segmentation* within the Bioconductor package “tilingArray” (http://www.bioconductor.org) [36], using 300 and 3,000 for the maxseg and maxk arguments, respectively. Since a one-to-one correspondence between the segments and the gene annotations does not exist (e.g., when several adjacent genes are not transcribed), tests of significance were carried out using the means of the probes that mapped fully to each annotated gene. The intensity threshold between the untranscribed and transcribed segments was determined by fitting a mixture model to the segment means using the “mclust” package from Bioconductor [36]. The significance of expression for each annotation was calculated using the binomial test on the signs of the differences between the probe intensities and the threshold [37]. The *p*-values were adjusted for multiplicity using the Benjamini–Yekutieli procedure from the “multtest” package of Bioconductor [36] with a false discovery rate of 0.1%.

### Luciferase reporter assay.

Regions of T. gondii DNA (Tables 2 and S1) were subcloned into pCR8-GW vector (Invitrogen, http://www.invitrogen.com), sequenced, and cloned into a Gateway vector expressing Firefly luciferase. Plasmid (50 μg) was co-transfected with 20 μg plasmid expressing Renilla luciferase under the control of the Tubulin promoter (both plasmids gift of M. W. White, Montana State University) following standard transfection protocols [38]. Luciferase assay was performed after 24 h using the Promega Dual-Luciferase Kit (http://www.promega.com) according to manufacturer instructions. Each assay was repeated three times in duplicate.

### Gene prediction criteria and ESTs databases.

Gene predictions were as described in Khan et al. [20]. The sequences corresponding to the CDS were extracted for a 650-kb region of the RH strain Chromosome 1b [20] and were set up as a BLAST database using the BLAST program downloaded from NCBI (http://www.ncbi.nlm.nih.gov/BLAST). We then used a perl script to blast the 88,535 EST sequences downloaded from the ToxoDB Web site (http://www.toxodb.org/download/release-3.3/EST/nuc) against the BLAST database. The e-value cut off of e-25 was considered significant.

## Supporting Information

Figure S1 Alignment of H3 and H4 Histones Sequences for T. gondii (Tg), P. falciparum (Pf), Homo sapiens (Hs), S. cerevisiae (Sc), and Drosophila melanogaster (Dm) H3 and H4 histones retrieved from the Histone Sequence Database (http://research.nhgri.nih.gov/histones) with a consensus sequence. Red letters represent the amino acids that are present in more than 90% of the sequences, and blue residues are present in at least 50% of the sequences.
(3.2 MB TIF)

Figure S2 T. gondii Nuclei Are Labeled with Antibody Recognizing Modified Histones Commercial antibodies (FITC; green) specific for histone modifications H3K4me3 (SF2A), H3K9ac (SF2B), and H4ac (SF2C) label the nucleus of the parasite (DAPI; blue). The phase-contrast images are presented in the right panel.
(2.7 MB TIF)

Figure S3 Promoter Assays of Selected Regions of Chromosome 1b The graph represents Firefly luciferase units normalized to Renilla luciferase counts (driven by constitutive tubulin promoter). Normalized counts for parasites transfected with Firefly luciferase under the control of (1) the bradyzoite-specific promoter *LDH2* (not expressed in the tachyzoite stage); (2) no DNA; (3) a region not predicted to be a promoter; (4) the probable promoter region of a predicted gene with neither “activation” histone peaks nor cDNA hybridization; (5) and (6) the “promoter” of the DNA-repair protein XCCR3 homolog (sense = 5 and antisense = 6), the only gene that had “activation” modified histone peaks but no cDNA hybridization; (7) the promoter of an expressed gene (positive cDNA hybridization) with peaks of modified histone enrichment.
(409 KB TIF)

Figure S4 Integration of ChIP-on-Chip and Expression Data Enables Improved Genome Annotation Distribution of identified H3K9ac peaks is illustrated in green. The moving average of the normalized expression data (cDNA hybridization signal) and significant peaks of expression are shown in black. The predicted genes are represented by the light blue boxes (up = sense; down = antisense; TgIb.1810c, TgIb.1800c, TgIb.1790c now represented by 25m0080, 25m0081 in ToxoDB version 4.1, http://www.toxodb.org). These CDS are marked from the predicted start and stop codon and do not reflect the likely presence of introns (as suggested by the discontinuous areas of cDNA hybridization seen on the microarray). The regions amplified by RT-PCR are boxed in red. The result of the RT-PCR is shown below. The presence (+) or the absence (−) of the reverse transcriptase enzyme in the RT reaction is indicated at the top of the gel.
(2.2 MB TIF)

Table S1 Summary of Significant ChIP-on-Chip Peaks with Associated Expression Data The 52 significant ChIP-on-Chip peaks identified by Chipotle software are listed with their position and size of peak (H3K4me3, H3K9ac, and H4ac peaks overlapped). From the results of the Chipotle and SignalMap analyses, significant hybridization compared with control for individual experiments and pooled data from all three experiments are indicated when using a threshold of 1 (log_2_; i.e., 2-fold) or a lower indicated threshold for regions in the proximity of each histone activation peak. Red + signs indicate hybridization present with only the lower threshold. RT-PCR was used to verify expression for the indicated genes. Fifty of 52 peaks had a region of cDNA hybridization within 1,000 bp. Peaks with corresponding tachyzoite ESTs are indicated. The significance of the means of the probes that map fully to the gene annotations and gene annotations with intensities above the threshold for transcription (identified using the segmentation analysis) are shown for peaks within 1,000 bp of an annotated gene (+++, *p* < 0.001; ++, *p* < 0.01; +, *p* < 0.05: (+), *p* < 0.1; ns, not significant). If more than one gene annotation was within 1,000 bp of the peak, then the closest annotation was tabulated. In six cases, the annotations are not within 1,000 bp of the peaks (x; distances noted) compared with two peaks that are not within 1,000 bp of transcription peaks (peaks 40 and 51) demonstrating the ability to improve predicted gene starts. Two verified (BD) and six possible (BD?) sequences able to drive reporter activity bidirectionally are indicated. Gene annotation numbers as described in [20].
(200 KB PDF)

### Accession Numbers

The European Bioinformatics Institute (http://www.ebi.ac.uk) accession numbers of genes and proteins used in this study are TgIb.1560c; TgIb.1570; TgIb.1580c; TgIb.1590; TgIb.1600; TgIb.1610c; TgIb.1620; TgIb.1630c; TgIb.1640; TgIb.1650c; TgIb.1660; TgIb.1670; TgIb.1680c; TgIb.1690c; TgIb.1700c; TgIb.1710; TgIb.1720c; TgIb.1730c; TgIb.1740; TgIb.1750c; TgIb.1760c; TgIb.1770c; TgIb.1780; TgIb.1790c; TgIb.1800c; TgIb.1810c; TgIb.1820; TgIb.1830; TgIb.1840; TgIb.1850c; TgIb.1860; TgIb.1870c; TgIb.1880; TgIb.1890; TgIb.1900c; TgIb.1910; TgIb.1920; TgIb.1930c; TgIb.1940; TgIb.1950c; TgIb.1960c; TgIb.1970; TgIb.1980c; TgIb.1990; TgIb.2000; TgIb.2010c; TgIb.2020; TgIb.2030; TgIb.2040; TgIb.2050; TgIb.2060; TgIb.2070c; TgIb.2071; TgIb.2080; TgIb.2090; TgIb.2100c; TgIb.2110; TgIb.2120c; TgIb.2130c; TgIb.2140c; TgIb.2150c; TgIb.2160c; TgIb.2170c; TgIb.2180c; TgIb.2190; TgIb.2200; TgIb.2210c; TgIb.2220c; TgIb.2230; TgIb.2240; TgIb.2250; TgIb.2260; TgIb.2270; TgIb.2280c; TgIb.2290; TgIb.2291; TgIb.2300; TgIb.2310; TgIb.2320c; TgIb.2330; TgIb.2340; TgIb.2350c; TgIb.2360c; TgIb.2370c; TgIb.2380c; TgIb.2390c; TgIb.2400; TgIb.2410; TgIb.2420; TgIb.2430; and TgIb.2440c.

GenBank dbEST (http://www.ncbi.nlm.nih.gov/entrez/query.fcgi?db=Nucleotide) accession numbers of ESTs used in this study are BG659482, CN197705, and CK737836.

Microarray data have been submitted to the Gene Expression Omnibus database (http://www.ncbi.nlm.nih.gov/projects/geo) under accession numbers GSM139203–GSM139216 and GSM139134–GSM139136; the number for the complete series is GSE7262.

## Abbreviations

ChIP: chromatin immunoprecipitation
dscDNA: double-stranded cDNA
EST: expressed sequence tag
HFF: human foreskin fibroblasts
ORF: open reading frame
RT-PCR: reverse transcriptase−PCR
