# Supplementary material for: Epigenomic Modifications Predict Active Promoters and Gene Structure in Toxoplasma gondii
Source: PLoS Pathog. 2007 Jun 8;3(6):e77. doi: 10.1371/journal.ppat.0030077 (PMC1891328; doi:10.1371/journal.ppat.0030077)
Supplement: Supplementary file 1 [file ppat.0030077.st001.pdf]

Gissot et al Supp Table 1

Peak	start	stop	size	ChIPOTle average ratio threshold=1				ChIPOTle average ratio threshold=0.6				SignalMAP threshold=1				SignalMAP threshold=0.8				TilingArray			EST	RT-PCR	Other
				GE1	GE2	GE3	Average	GE1	GE2	GE3	Average	GE1	GE2	GE3	Average	GE1	GE2	GE3	Average	P-value	>threshold	Gene number			
1	1220	2650	1430	+	+	+	+	+	+	+	+	+	+	+	+	+	+	+	+	ns	+	1	+	+	
2	19750	20850	1100	+	+	+	+	+	+	+	+	+	+	+	+	+	+	+	+	+++	+	3	+		
3	26150	28000	1850	+	+	+	+	+	+	+	+	+	+	+	+	+	+	+	+	+++	+	5	+		
4	50350	52750	2400	+	+	+	+	+	+	+	+	+	+	+	+	+	+	+	+	+++	+	6	+		
5	53500	55050	1550	-	+	+	+	-	+	+	+	+	+	-	+	+	+	+	+	x	x	7	-	+	2 kb away
6	74400	76200	1800	-	+	+	+	-	+	+	+	+	+	+	+	+	+	+	+	++	+	10	+		
7	78000	80600	2600	+	+	+	+	+	+	+	+	+	+	+	+	+	+	+	+	+++	+	11	-		
8	116750	118350	1600	+	+	+	+	+	+	+	+	+	+	+	+	+	+	+	+	x	x	14	+		1.5 kb away
9	129771	131521	1750	+	+	+	+	+	+	+	+	+	+	+	+	+	+	+	+	ns/+	+/+	15,16	+		BD
10	152621	154122	1501	-	-	-	-	-	+	+	+	-	+	-	-	+	+	+	+	ns	+	18	+	+	
11	155272	157022	1750	-	+	+	+	+	+	+	+	+	+	+	+	+	+	+	+	+++	+	19	-		
12	167622	169872	2250	+	+	+	+	+	+	+	+	+	+	+	+	+	+	+	+	+++	+	20	+		
13	179322	180622	1300	+	+	+	+	+	+	+	+	+	+	+	+	+	+	+	+	ns	+	23	-		BD?
14	200872	204024	1152	+	+	+	+	+	+	+	+	+	+	-	+	+	+	+	+	+++	+	24/25/26	+	+	BD?
15	210624	212074	1450	+	+	+	+	+	+	+	+	+	+	+	+	+	+	+	+	ns	+	28	+		
16	214424	215974	1550	-	-	-	-	-	+	+	+	-	-	-	-	-	+	+	+	ns	-	29	+	+	
17	219824	221574	1750	-	+	+	+	+	+	+	+	+	+	+	+	+	+	+	+	ns/+	+/+	30/31	+		BD?
18	231824	233624	1800	+	+	+	+	+	+	+	+	+	+	+	+	+	+	+	+	++	+	33	+		BD with noncoding
19	261028	262278	1250	+	+	+	+	+	+	+	+	+	+	+	+	+	+	+	+	+++	+	36	+		
20	268932	271432	2500	+	+	+	+	+	+	+	+	+	+	+	+	+	+	+	+	++	+	37	+		
21	281332	282682	1350	+	+	+	+	+	+	+	+	+	+	+	+	+	+	+	+	+++	+	38	+		
22	283282	284533	1251	+	+	+	+	+	+	+	+	+	+	+	+	+	+	+	+	+++	+	39	-	+	
23	302284	303684	1400	+	+	+	+	+	+	+	+	+	+	+	+	+	+	+	+	x	x	40/41	-		2 kb away
24	304934	306036	1102	+	+	+	+	+	+	+	+	+	+	+	+	+	+	+	+	+++	+	42	+		
25	314386	315936	1550	+	+	+	+	+	+	+	+	+	+	+	+	+	+	+	+	+++	+	43	+		
26	316736	318094	1358	+	+	+	+	+	+	+	+	+	+	+	+	+	+	+	+	+++	+	44	-		
27	335894	338044	2150	+	+	+	+	+	+	+	+	+	+	+	+	+	+	+	+	+++	+	45	-		
28	347394	349744	2350	+	+	+	+	+	+	+	+	+	+	+	+	+	+	+	+	++	+	46	+		
29	350444	351994	1550	-	+	+	+	+	+	+	+	+	+	+	+	+	+	+	+	+++	+	47	+		
30	352294	355244	2950	+	+	+	+	+	+	+	+	+	+	+	+	+	+	+	+	+++	+	47	+		
31	365394	367444	2050	+	+	+	+	+	+	+	+	+	+	+	+	+	+	+	+	+++	+	51	+		
32	375494	377444	1950	+	+	+	+	+	+	+	+	+	+	+	+	+	+	+	+	x	x	x	+		no annotated gene
33	383878	386077	2199	+	+	+	+	+	+	+	+	+	+	+	+	+	+	+	+	+++/ns	+/+	52,53	+		
34	386427	388927	2500	+	+	+	+	+	+	+	+	+	+	+	+	+	+	+	+	+++	+	54	-	+	
35	404779	405979	1200	+	+	+	+	+	+	+	+	+	+	+	+	+	+	+	+	+++	+	55	-	+	
36	452429	454379	1950	+	+	+	+	+	+	+	+	+	+	+	+	+	+	+	+	+++	+	63	+		
37	458279	461162	2883	+	+	+	+	+	+	+	+	+	+	+	+	+	+	+	+	++	+	64	+		BD?
38	465182	466682	1500	+	+	+	+	+	+	+	+	+	+	+	+	+	+	+	+	+++	+	66	+		
39	491428	493278	1850	+	+	+	+	+	+	+	+	+	+	+	+	+	+	+	+	x	x	67	+		1.5 kb away
40	502079	503779	1700	+	+	+	+	+	+	+	+	+	+	+	+	+	+	+	+	x	x	68	+	+	4 kb away
41	504979	507029	2050	+	+	+	+	+	+	+	+	+	+	+	+	+	+	+	+	+++	+	69	+		
42	518329	519519	1190	-	-	+	-	+	+	+	+	-	+	+	+	+	+	+	+	+	+	70	-	+	
43	528629	530579	1950	-	-	+	-	+	+	+	+	-	+	+	-	+	+	+	+	+++	+	71	-	+	
44	537841	539555	1714	-	+	+	+	-	+	+	+	+	+	+	+	+	+	+	+	+	+	72	+		
45	543105	545515	2410	-	+	+	+	+	+	+	+	+	+	+	+	+	+	+	+	+++	+	75	+		BD?
46	557455	558955	1500	+	+	+	+	+	+	+	+	+	+	+	+	+	+	+	+	(+)	+	76	+		BD?
47	572755	575505	2750	+	+	+	+	+	+	+	+	+	+	+	+	+	+	+	+	+++/+++	+/+	79,80	-/+		
48	579505	581155	1650	+	+	+	+	+	+	+	+	+	+	+	+	+	+	+	+	+++	+	81	+		
49	601755	603405	1650	+	+	+	+	+	+	+	+	+	+	+	+	+	+	+	+	+++	+	87	+		
50	620680	622930	2250	+	+	+	+	+	+	+	+	+	+	+	+	+	+	+	+	+++	+	88	+		
51	633900	635400	1500	-	-	-	-	-	-	-	-	-	-	-	-	-	-	-	-	ns	-	89	-	-	DNA repair
52	637840	639300	1460	+	+	+	+	+	+	+	+	+	+	+	+	+	+	+	+	+++	+	90	-	+	
